# Genetic and Bioinformatic Strategies to Improve Diagnosis in Three Inherited Bleeding Disorders in Bogotá, Colombia

**DOI:** 10.3390/genes12111807

**Published:** 2021-11-18

**Authors:** Juliana Lago, Helena Groot, Diego Navas, Paula Lago, María Gamboa, Dayana Calderón, Diana C. Polanía-Villanueva

**Affiliations:** 1 Laboratorio de Genética Humana, Universidad de Los Andes, Bogotá 111711, Colombia; j.lago@uniandes.edu.co (J.L.); hgroot@uniandes.edu.co (H.G.); dl.navas@uniandes.edu.co (D.N.); 2 Department of Basic Sciences, Kyushu Institute of Technology, Kitakyushu 804-8550, Japan; paula@sozolab.jp; 3 Laboratorio de Referencia en Hemostasia, Bogotá 110231, Colombia; mcgamboa@labhemostasia.com; 4 Corporación Corpogen, Universidad Central, Bogotá 110311, Colombia; dcalderonm@ucentral.edu.co

**Keywords:** hemophilia, von Willebrand Disease (VWD), high-resolution melting (HRM), genetic diagnosis, Python code, denaturation domain

## Abstract

Inherited bleeding disorders (IBDs) are the most frequent congenital diseases in the Colombian population; three of them are hemophilia A (HA), hemophilia B (HB), and von Willebrand Disease (VWD). Currently, diagnosis relies on multiple clinical laboratory assays to assign a phenotype. Due to the lack of accessibility to these tests, patients can receive an incomplete diagnosis. In these cases, genetic studies reinforce the clinical diagnosis. The present study characterized the molecular genetic basis of 11 HA, three HB, and five VWD patients by sequencing the *F8, F9*, or the *VWF* gene. Twelve variations were found in HA patients, four in HB patients, and 19 in WVD patients. From these variations a total of 25 novel variations were found. Disease-causing variations were used as positive controls for validation of the high-resolution melting (HRM) variant-scanning technique. This approach is a low-cost genetic diagnostic method proposed to be incorporated in developing countries. For the data analysis, we developed an accessible open-source code in Python that improves HRM data analysis with better sensitivity of 95% and without bias when using different HRM equipment and software. Analysis of amplicons with a length greater than 300 bp can be performed by implementing an analysis by denaturation domains.

## 1. Introduction

Inherited bleeding disorders (IBDs) are a group of conditions where deficiencies in plasma proteins, involved in blood coagulation, lead to increased risk of bleeding [1]. People suffering from bleeding disorders may experience spontaneous or increased tendency to bleed, particularly around invasive procedures. However, bleeding symptoms vary according to the severity of the disease [2]. Hemophilia A (HA), hemophilia B (HB), and von Willebrand Disease (VWD) are the most common IBDs associated with a lack of clotting factors (blood coagulation factor VIII (F8), factor IX (F9), and von Willebrand factor (VWF), respectively). Although they are rare diseases, their correct diagnosis is an important component of clinical management as it may inform treatment decisions and carrier status [3,4].

Diagnosis is usually performed through factor assays specific for coagulation proteins and genetic testing where available. However, unclear diagnosis in many cases may occur, as test results suffer from extreme variability, in part because of the wide range of methods, reagents, and instruments, and the lack of access to genetic testing [5]. This is evidenced by the differences in reports of prevalence. Data sourced from the World Bank Group show that 2639 individuals are diagnosed with HA, 591 with HB, and 2770 with VWD in Colombia. However, according to the report from the high-cost account published in Colombia in 2019 [6], 1916 individuals are diagnosed with HA, 415 with HB, and 1444 with VWD. These data suggest the lack of clarity in the exact prevalence in the country and exemplify the need to implement genetic studies that support a more precise diagnosis. It is critical for healthcare planning that reliable prevalence data of these diseases are available, especially in developing countries such as Colombia. With improved data, healthcare resources can be better managed and allocated [7].

Genetic testing for bleeding diseases is possible but costly, and therefore usually unavailable in developing countries such as Colombia. Therefore, variant-scanning techniques would be beneficial for the identification and study of these disease-causing variations [8,9,10,11]. The high-resolution melting (HRM) technique is one such variant-scanning technique consisting of the quantitative analysis of the melt curves of product DNA fragments following PCR amplification [12]. This technique has had a great impact in the clinical field, mainly in the diagnosis of diseases [8,10,11,13,14,15,16,17]. Data produced through HRM technology are analyzed in commercial HRM software packages, however, these packages usually require manual manipulation to select the starting and ending point of the melting curve, causing subjective and non-replicable analysis, as the results may depend on the person doing the analysis. Moreover, when long fragments are analyzed, the DNA separates in multiple steps, causing differences in the curve, called domains, which cannot be analyzed by commercial software. To analyze this, multiple PCR amplifications are needed, elevating the costs of the technique.

In this paper we found twelve variations in HA patients, four in HB patients, and nineteen in WVD patients, and validated them through HRM. We also propose an open-source code to analyze HRM curves including domain analysis. The code improves the efficiency of HRM analysis as it eliminates manual operation and provides a cost-effective method to evaluate gene variations for bleeding disorders, as it can be used to analyze long fragments from PCR amplifications. We use this code to analyze variations found in patients diagnosed with HA or HB. Fourteen patients and 16 controls were studied with the code. HRM data analysis had a sensitivity of 95%.

## 2. Materials and Methods

### 2.1. Study Sample

The study was approved by the Ethics Committee of Los Andes University, and the methods were applied following the approved guidelines. Written informed consent was obtained from all participants. Seven patients and four carriers with HA, one patient and two carriers with HB, and five patients with VWD were selected from 20 unrelated families. Recruited patients attended the Colombian League of Hemophiliacs and Other Blood Deficiencies in Bogotá, Colombia. All patients included in the study presented a supported medical history accompanied by hematological diagnoses. A retrospective analysis of the clinical history of each family member was performed to evaluate each coagulopathy score. The following laboratory tests were evaluated: thrombin, prothrombin and thromboplastin time, ristocetin-cofactor, FVIII, VWF, and FIX9 activity, respectively.

Inclusion criteria: Colombian male and female patients of legal age with a confirmed clinical diagnosis of HA, HB, or VWD. Some hemophilia patients were underage; in these cases, blood samples were extracted from mothers as obligate carriers to avoid health complications. Carriers are all women with at least one hemophilic male in their maternal pedigree. Normal FVIII and FIX plasma levels did not rule out the carrier status of a female since FVIII or FIX levels show considerable variability, and normal levels do not always represent non-carrier status. The phenotype characteristics of patients are listed in Tables S1–S5.

Exclusion criteria: Patients with a diagnosis of acquired hemophilia A or any other bleeding disorder or unknown hemostatic defects; patients not of legal age.

Eight healthy males and eight healthy females were included as controls for HRM results validation.

Inclusion criteria: Colombian male and female subjects of legal age.

Exclusion criteria: Subjects with a diagnosis of acquired hemophilia A or any other bleeding disorder or unknown hemostatic defects; patients not of legal age.

### 2.2. DNA Extraction

Blood samples were taken at the Haemostasis Reference Laboratory in Bogotá, Colombia. Samples were transported in the first 4 h to the Human Genetics Laboratory of Los Andes University. DNA was extracted from peripheral blood leukocytes using the proteinase K and phenol–chloroform extraction method [18]. The sample concentration was determined by NanoDrop™.

### 2.3. Genetic Analysis of F8, F9, and VWF Genes

In patients with HA diagnosis, the presence of intron 22 and intron 1 inversion was evaluated. Intron 22 inversion detection was performed according to the protocol followed by Polanía et al. [19]. Intron 1 inversion was evaluated through the PCR protocol described by Bagnall et al. (2012) [20]. The PCR-multiplex temperature conditions for inversion 1 included an initial denaturation at 95 °C for 2 min, followed by 30 cycles composed of denaturation at 94 °C for 30 s, alignment at 63 °C for 45 s, and an extension at 72 °C for 2 min. Subsequently, a 1% agarose gel was run at 70 V for 60 min to observe and analyze the expected products. TrackIt 1 Kb plus DNA ladder (Invitrogen, USA was used.

To detect gene variations, forward and reverse primers were designed for the *VWF* and *F9* genes (*F9* ID: 2158 and *VWF* ID: 7450) (shown in Table 1) based on the NCBI reported sequence. The possible variations present in *VWF* exons 17–25 (VWD type 2N) and 28 (VWD types 2A, 2B, and 2M) were genetically evaluated to confirm or rule out VWD type 2 and type 1 diagnosis [21]. WVD type 3 was discarded considering the clinic of patients. All primers were designed and validated with the help of the NCBI tool [22], and the Genome Browser of Santa Cruz University of California [23]. *F8* primer sequences were obtained from the Hadb database [19].

Samples were amplified under the following PCR conditions: 95 °C for 2 min, followed by 30 cycles (that varied according to each primer pair, see Table 1) that, for the case of *F8* primers, included 94 °C for 30 s, 59 °C for 45 s, and 72 °C for 90 s. The final extension was 72 °C for 5 min. Finally, electrophoresis was performed to evaluate the expected sizes for each amplicon in a 2% agarose gel at 70 V for 60 min. Subsequently, the PCR products were sequenced by the Sanger method in the ABI prism 3500^®^ genetic analyzer.

### 2.4. Variation Effect Analysis on F8, F9, and VWF Proteins

Each patient’s exon sequence was aligned with the consensus sequence provided by NCBI (*F8* ID: 2157, *F9* ID: 2158, and *VWF* ID: 7450) in Chromas v2.6, Geneious v4.5, and BioEdit v7.0.5.3. To know the possible effect of these variations on the biological function of the evaluated proteins, five predictors were used: SIFT, PolyPhen-2 [24], Mutation Taster [25], variant effect predictor (Ensembl) [26], and PhD-SNP [27]. Specialized databases were checked for previously reported gene variants, such as SNPdb, EAHAD Coagulation Factor Variant Database [28], and Human Gene Mutation Database (HGMD).

Among the criteria used to analyze the results obtained from the different predictors are the following: major criteria: finding null variants; strong criteria: amino acid change previously established as a pathogenic variant, in vitro or in vivo established pathogenic variant, functional studies supporting a deleterious effect on the gene product, prevalence of the variant in affected individuals compared to controls or evidence to be pathogenic; moderate criteria: located in a mutational and/or critical hotspot point and well-established functional domain, absence of the variant in control groups, for recessive disorders that are detected in trans with a pathogenic variant, changes in protein length as a result of in-frame deletions or insertions in a region without repeats, amino acid changes determined to be pathogenic, moderate pathogenicity, de novo variant but without confirmation of paternity or maternity; supporting criteria: multiple bioinformatic predictors support a detrimental effect, the patient’s phenotype or family history is highly specific for a disease with a single genetic etiology, when a reliable source reports it as a pathogen without there being any functional studies.

### 2.5. High-Resolution Melting Analysis

After fully screening each patient’s genotype with routine methods (Sanger sequencing), exons with variations were used as positive controls for the HRM technique validation. Analyses were performed under the Roche protocol for variation detection (LightCycler^®^ 96 System User Training Guide, v2.0) in a real-time PCR LightCycler^®^ 96 System. MeltDoctor ™ HMR (Thermo Fisher) reagents were used, along with the respective primers for each reaction, DNA template (25 ng/µL), and water. After real-time PCR, a dissociation of the amplification was performed with a fusion step. The thermal profile corresponds to a preincubation step of 95 °C for 10 min with a ramp of 4.4 °C/s. This step was followed by a 3-step amplification that included: denaturation at 95 °C for 15 s, hybridization at a specific temperature according to each exon for 20 s, and amplification at 72 °C for 20 s, for 45 cycles. This was followed by a high-resolution melting step at 95 °C for 60 s, 40 °C for 60 s with a ramp of 2.2 °C/s, 65 °C for 1 s, and 97 °C for 1 s with a ramp 0.04 °C/s with maximum 25 readings/°C corresponding to the highest number of fluorescence readings per degree.

For male DNA analysis, hemizygous DNA was mixed with healthy male DNA. This mixture improves the detection performance, as previously reported [8,29,30].

### 2.6. High-Resolution Melting Data Analysis

Values obtained from the DNA fusion curves were analyzed with the High-Resolution Melt software for LightCycler 96 System Software and, to avoid manual analysis of the HRM melting curves, we developed an algorithm in Python based on the algorithm proposed by Li et al. (2016) [30]. We extended the algorithm to allow for domain identification and analysis and analysis against multiple control curves considering the possible deviations of normal curves. Instead of using predefined angles to detect the start and end of the melting region, we used the second derivative of the melting curve to detect minimum points and peaks. The code is available at: https://gitfront.io/r/user-5547184/d3ec1062bc7d0d2e3039b74770330c06f510d65e/hrm-analysis/, accessed on 20 August 2021.

#### 2.6.1. Domain Identification

Melting large amplicons, such as exons with a size greater than 300 bp, can result in multiple melting domains, which complicate the HRM analysis. Usually, the solution for this is to create smaller amplicons, but this can increase the cost of the analysis as multiple samples are needed. Instead, we analyzed each domain separately. A domain is identified as a peak in the derivative curve. A peak in a curve is detected when its derivative changes from negative to positive. To identify domains in the melting curve, we applied a Savitsky–Golay filter using the savgol_filter function of the scipy signal library for Python to identify the second derivative of the curve. We then identified the points where it changes its sign from positive to negative. These are considered the peaks in the first derivative of the melting curve. Peaks with values below a threshold were not considered a domain for the analysis, as they were found to be noise. The threshold value was set to 0.10 by analyzing the number of domains in most curves. Each of the resulting peaks represented a domain in the melting curve. For each domain, we then identified its melting region.

#### 2.6.2. Domain Melting Region Identification and Analysis

To identify the melting region of each domain, we found the minimum points in the second derivative. These are identified as the points where the sign changes from positive to negative. The start of the melting region was set 2 degrees before the start point of the domain and the end of the melting region as 2 degrees after the end of the domain, as suggested by Li et al. (2016) [30]. For each domain, curve normalization and background subtraction were performed following the procedure suggested by Li et al (2016) [30]. The melting temperature corresponded with the peak of the domain found previously.

#### 2.6.3. Difference Plot

The preferred data display for high-resolution amplicon melting analysis is a difference plot which shows the difference between the analyzed curve and the mean curve of the set of controls. One difference plot for each domain was created. The median was chosen instead of the mean curve because the median curve is less affected by outliers. We also plotted the standard deviation of the median curve (shown as a gray area) to represent the variability of the set of controls.

## 3. Results

### 3.1. Genetic Analysis of F8, F9, and VWF Genes

**Figure 1:**
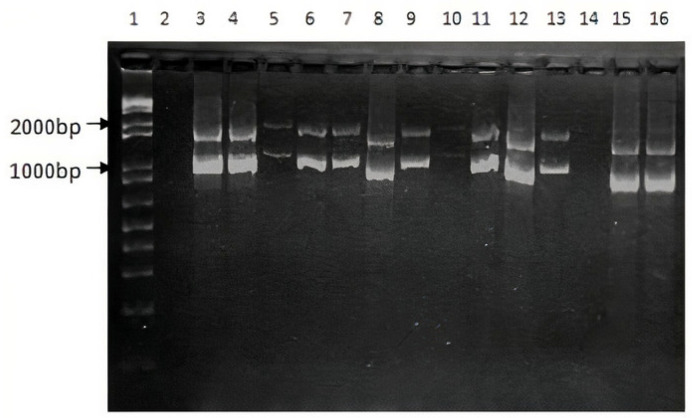
PCR test for the F8 int1h-related inversion. Lane 1 corresponds to 1Kb ladder. Lane 2: Negative control. Amplification products for HA patients from HA1 to HA11 (Lanes 3 to 13). Amplification products for 2 healthy subjects (Lanes 15 and 16).

Table 2 reports the main variations found in the study sample associated with the diagnosis of each patient as well as the effect of each variation in the F8, F9, or VWF protein according to the results obtained by the different predictors. Inversion 1 (Figure 1) and inversion 22 diagnosis was ruled out in all HA patients. Twelve variations were found in HA patients, four in HB patients and 19 in WVD patients. From these variations, a total of twenty-five novel variations were found. Table 3 summarizes and classifies the variations found in the population under study, according to their effect on the protein and their report in the literature. To our knowledge, these new variants have not been reported in other previous studies carried out in the Colombian population [10].

### 3.2. Development of an Open-Source Code in Python for HRM Data Analysis

**Figure 2:**
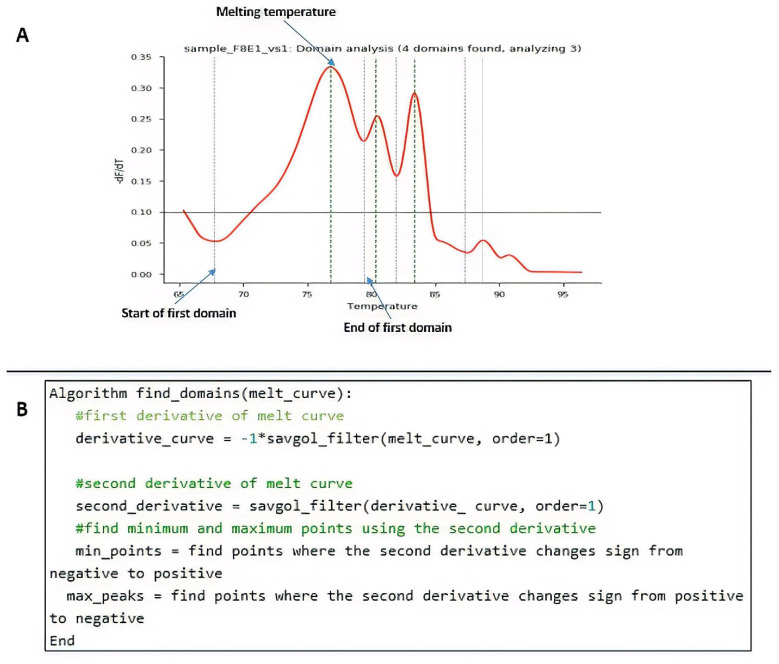
Procedural programming scripts developed in Python. (**A**) Example of peak identification through the algorithm. (**B**) Pseudo-algorithm of the process.

HRM analysis was enriched through an open-source code in Python. Steps included the following: to extract each domain, the melting curve was preprocessed using the Savistzky–Golay filter to obtain the first derivative curve of each sample. Using this derivative curve, all peaks were identified by finding the point where the second derivative changes from negative to positive (see Figure 2A which shows four peaks). Peaks with values below a threshold were not considered a domain for the analysis, as they were found to be noise. The threshold value was set to 0.10 by analyzing the number of domains in most curves. Each of the resulting peaks represented a domain in the melting curve. For each domain, the start and end of the melting region were found by finding the minimum points of the curve that enclosed the peak. To do this, the points found in the second derivative where the value changes from positive to negative (minimum points of the curve). A pseudo-algorithm of this process is shown in Figure 2B.

After finding the domains, each of them was analyzed as a new melting region following the steps in the procedure by Li et al. (2016) [30]. The start of the melting region was set 2 degrees before the start point of the domain and the end of the melting region as 2 degrees after the end of the domain, as suggested by the authors. For each domain, curve normalization and background subtraction were performed, as well as finding the melting temperature. The melting temperature corresponded with the peak of the domain found previously.

Finally, for the difference plot construction, one difference plot for each domain was created, comparing with the median curve for the same domain of a set of control experiments. Each melting curve in the dataset was subtracted from the median curve. The median was chosen instead of the mean curve as Li et al. (2016) [30] did because the median curve is less affected by outliers.

### 3.3. High-Resolution Melting Technique Validation

**Figure 3:**
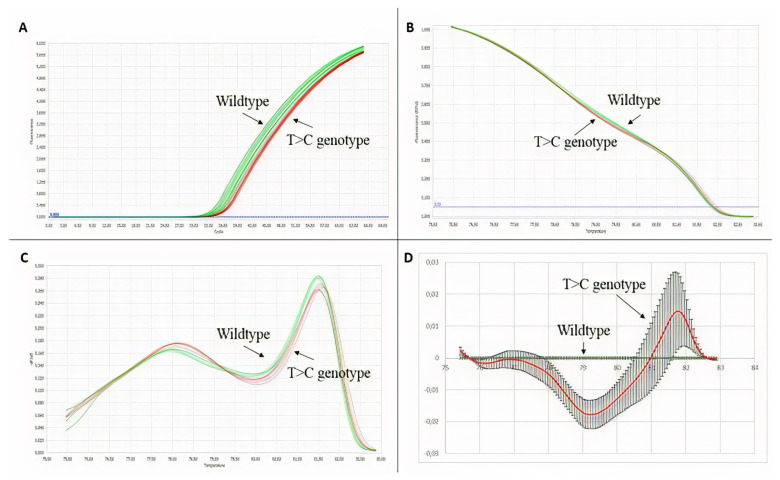
HRM technique validation results. In green wildtype, genotype samples, and in red, T > C genotype samples. (**A**) Amplification plot. (**B**) Normalized melting curves. (**C**) Normalized melting peaks. (**D**) Difference plot.

In a preliminary way, we wanted to evaluate the pertinence of the HRM technique in identifying point variations. For this purpose, a point variation (c.440T > C) found on exon 4 of the *F8* in two HA patients was selected as a positive control. Data were analyzed with the commercial software from Roche. As shown in Figure 3, it is evident that despite the variation of the data, the average behavior of the wildtype and mutant curves is different. Differences were judged significant if the curves of a mutated amplicon were found with similar values outside the range of normality.

**Figure 4:**
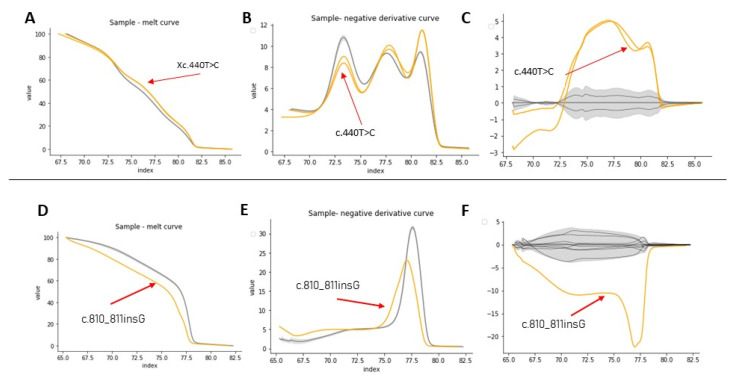
HRM analysis of exon 4 from samples Af3A/Af4A (**A**–**C**) and exon 7 from sample Af1B (**D**–**F**). In yellow, the positive control, in gray, the healthy controls. (**A**–**D**) Denaturation curves, X axis = temperature (°C), Y = normalized fluorescence (RFU). (**B**–**E**) Negative derivative plot (-dF/dT), (X axis = temperature (°C), Y = normalized fluorescence (RFU)). (**C**–**F**) Plot of differences (X axis = temperature (°C), Y = normalized fluorescence (RFU)).

This preliminary validation with the point variation was extended to the other variations previously found by Sanger sequencing from the other patients with HA and HB to confirm the effectiveness of the technique in scanning variations. To improve our data analysis, denaturation curves were analyzed using Python programming code. By scanning variations in the exons of patients diagnosed with HA or HB, curve profiles other than the normal type were found. Table 4 summarizes HRM data results, where 21 out of a total of 22 variations were validated. Figure 4A–C show the HRM analysis of variation c.440T > C on exon 4 of the F8 with the Python code. Better differentiation of the samples can be evidenced for healthy controls in contrast to the graphs obtained with the commercial software shown in Figure 3.

For exons with a size less than 300bp, the analysis by HRM using the code was successful. For example, Figure 4D–F show the HRM analysis for exon 7, that has 150 bp, of sample Af1B. This sample presented a nucleotide insertion and shows a clearly differentiable curve concerning samples from healthy individuals. Analysis of the variants found in samples af2A, Am2a, Am3a, Am5a, Am6a from exon 24 was also successful (Figures S2 and S3), however, in these samples, it was necessary to discriminate the analysis by sex to obtain better results. It was not possible to identify a different curve profile for exon 6 from sample Af2A, because the curve of the negative derivative plot was within the variation pattern of the curves from healthy controls, probably because of the position of the variation in the sequence.

**Figure 5:**
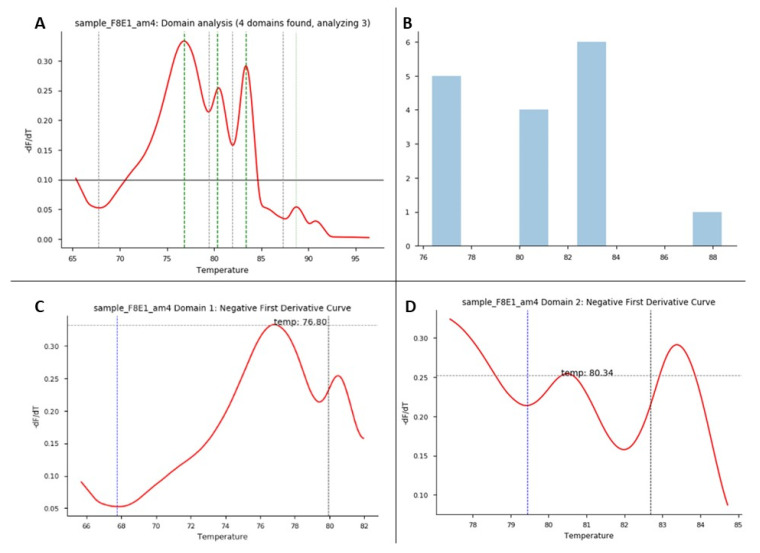
HRM domain analysis. HRM domain analysis for exon 1 of *F8*: this experiment consisted of 1 patient as a positive control and 3 healthy controls. (**A**) Domain analysis plot: for each sample/exon, Python shows the number of melting domains identified; in this example, the figure shows 4 identified domains in the positive control sample. (**B**) Domain analysis plot: for each data run, this graph shows the number of domains identified in the samples run in the experiment between some temperatures that correspond to the identified melting regions. (**C**,**D**) Plots correspond to negative first derivative plot for each specific domain identified in each sample. Each graph shows the melting temperature the program identifies for each domain.

**Figure 6:**
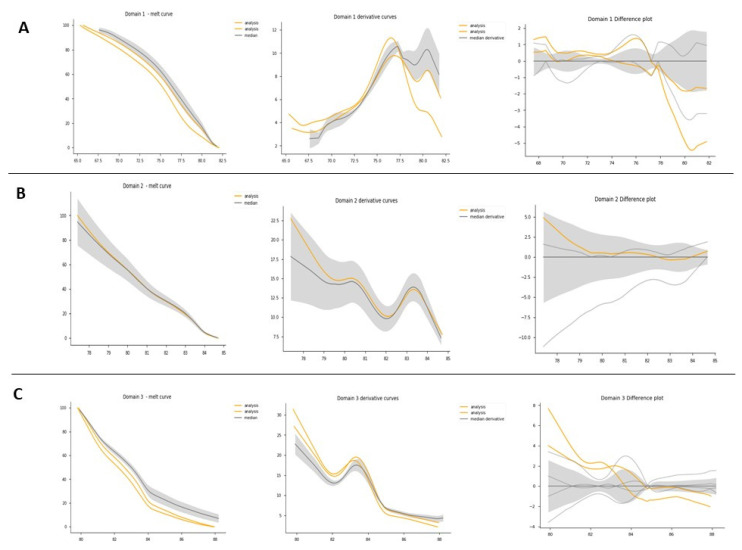
HRM domain analysis. HRM domain analysis for exon 1 of *F8*: this experiment consisted of 1 patient as a positive control and 3 healthy controls. (**A**) All samples domain 1 analysis: from right to left: melt curve analysis plot: normalized melting curves of each sample are presented by domain. Derivative curves: identify the Tm between each domain. Difference plot: the baseline for the difference plot construction consisted of a composite median reference curve (in gray) of all wildtype curves to facilitate clusters around the horizontal axis. (**B**) All samples domain 2 analysis. (**C**) All samples domain 3 analysis.

**Figure 7:**
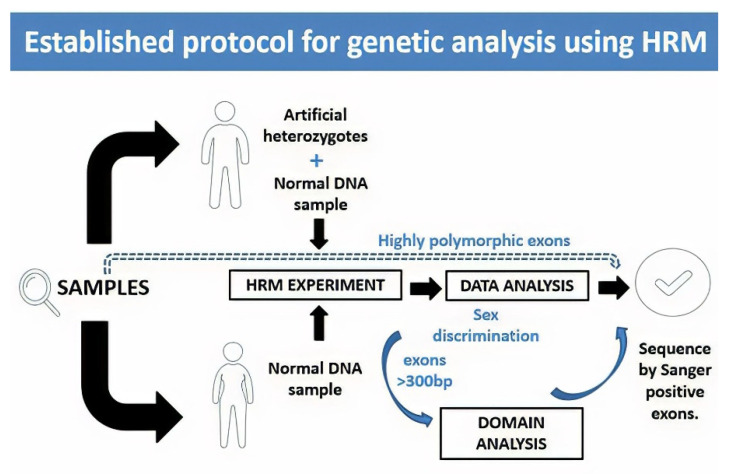
Proposed protocol for genetic analysis using HRM. In this protocol, we propose to take the DNA samples to carry out the HRM test and, in the case of men, an artificial heterozygote should be included in the test run. In the case of exons that are reported as highly polymorphic, it is recommended to sequence without a previous scan by HRM, as they will probably give positive results. Once the data from the qPCR equipment are obtained at the time of the analysis, we recommend discriminating the samples by sex, and in the case of exons with a size greater than 300 bp, to include an analysis by domains. Samples that are positive through these analyses must be confirmed by Sanger sequencing.

Exons with a length greater than 300 bp presented melting curves with several melting domains. To improve sensitivity, analysis by denaturation domains was implemented [13]. The analysis was performed in two steps, first studying the overall amplicon and then each domain. Figure 5 shows the domain analysis for a point variation (g.4266C > T) on exon 1 of the *F8* found in one HA patient. For this experiment, three healthy subjects were used as controls for validations. Figure 5 shows the identification of each domain within a sample, while Figure 6 shows the HRM analysis by domain. In this experiment, Figure 6 shows the healthy controls in gray and the shadow shows the mean with its deviation. The analysis (shown in yellow) corresponds to the positive control, where we have the DNA of the patient that corresponds to an affected male and the same patient as an artificial heterozygote. In Figure 6B, the three graphs (melt curve, negative derivative plot, and difference plot) obtained for the analysis of domain 2 are shown, and in this case, we see that in this domain there are no differences between healthy controls and positive controls. Therefore, in that region of the amplified fragment, we can deduce that there are no differences in the DNA sequence. On the other hand, domains 1 and 3 show us differences in both the patient’s DNA and the artificial heterozygote of the same patient. In this case, when finding these differences in a blind test or with an unknown sample, that exon of that patient should be sequenced to identify the specific variation. Figure 6C shows the greater differences between samples and healthy controls. As seen in Figure 5B, in all the samples analyzed in this experiment, domain 3 was detected, and as evidenced in Figure 6C, it is the most informative domain to detect differences in this experiment. Finally, we propose a protocol for molecular analysis using HRM that supports the clinical diagnosis of patients with hemophilia, shown in Figure 7.

## 4. Discussion

### 4.1. Genetic Analysis of F8 Gene

Genetic analyses in all cases were performed under the HGVS Recommendations for the Description of Sequence Variants [31]. The intron 22 and 1 inversions (Inv22 and Inv1) are the most frequent molecular alterations found in severe HA patients with a frequency of 45–50% and 0.5–5%, respectively. However, in a study carried out in Colombia by Yunis et al. (2018), inversion 22 was detected in 14/33 male patients (42.4%), and inversion 1 was detected in 3/33 [10]. These results are consistent with our study since it must be considered that, in total, we had 11 patients with hemophilia A, from which four were men classified as severe, three men classified as mild, and four carriers. In this scenario, it is consistent to find negative results of inversion assays.

**Figure 8:**
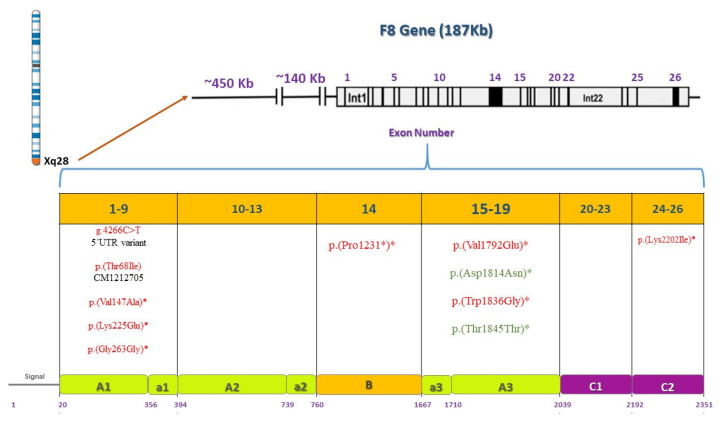
Pathogenic or benign variations found by *F8* gene and protein domain. *F8* is located on the X chromosome, 26 exons are shown and their corresponding domains and motifs in the F8 protein. Deleterious variations appear in red and benign variations in green. Asterisks indicate novel variations found.

Patient Af1A is an HA carrier with low F8 levels (24.3%) (Tables S1 and S2). This patient presents a c.3690_3691insG in exon 14 (Figure 8). The variation results in a truncated protein, so it should have clear functional consequences. Patient Af2A is also an HA carrier, despite having normal FVIII levels. This phenomenon has been observed in other studies where bleeding tendency is also observed in some hemophilia A carriers with normal factor VIII levels [32]. This patient presents variations c.673A > G and c.6605A > T. Supporting criteria for these variations include multiple bioinformatic predictors that support a detrimental effect and the patient’s family history is highly specific for the disease.

The only way to ascertain the carrier status is through a molecular diagnosis of the causative mutation in F8 or F9 as we did. Gene analysis is the gold standard for carrier diagnosis as it identifies undetected female carriers. Despite the clear advantage of next-generation sequencing in various settings, Sanger sequencing remains a better option for genetic testing and diagnosis of hemophilia carriers. There may still be a variety of unknown mutations of F8 and F9 target genes as our research and Shinozawa et al. (2021) reported [4]. Any new variation found by NGS must still be validated by Sanger sequencing. It is clearly seen that corroborating the carrier status of women, whose factor measurements may be normal, is necessary through genetic analysis.

Variation c.6605A > T was also found in patient Am6A whose phenotype is mild (Tables S1 and S2). In these two cases, both patients present the same variation, however, each variation’s phenotypic penetrance and expressivity vary due to the different combinations of modifying alleles that are present in one genetic background versus another. In addition, the penetrance of some pathogenic genotypes is known to be age and/or sex dependent. Variable penetrance may also reflect the action of unlinked modifier genes, epigenetic changes, or environmental factors. Some examples of pathogenic microlesions whose penetrance has been found to be modulated by allelic SNPs are provided by Cooper et al. [33]. In the study reported by Venceslá et al. (2010), three consanguineous sisters presented the same homozygous variation. However, two sisters had moderate bleeding due to a known mutation, and one of the sisters had no bleeding history [34].

Patient Am1A, with a mild phenotype, presented variation c.5375T > A which was also predicted as pathogenic. Patient Am2A is a severe HA case. Variation c.203C > T is reported by the HGMD (CM105565) as a known disease variation (Figure 8). This variation was also found in a severe HA patient in Sweden [35]. This patient has a positive inhibitor test result, and it is known that patients who develop inhibitors usually have significant changes in the *F8* sequence [36]. Patients Af3A and Af4A are both HA carriers with variation c.440T > C. Both patients have sons with severe phenotypes. This variation is a known disease variation reported by the HGMD (CM021585) (Figure 8). Patient Af3A is a symptomatic carrier with bleeding tendency. This phenomenon has also been observed in some hemophilia A carriers with normal factor VIII levels as in this case and requires further investigation (Tables S1 and S2) [32,37,38,39]. Shinozawa et al. (2021) mention the impact of age and blood group on FVIII:C level [4]. Biguzzi et al. (2021) reported that VWF:Ag and FVIII:C increase with age [40]. Carriers of a non-O blood group present a steeper increase in VWF:Ag and FVIII:C with age, which is mediated by acquired factors. Additionally, it is consistent with Af3A’s blood type B and her age. Many studies have reported that there are great inter- and intraindividual variations in coagulation markers in women due to different physiological conditions such as age, ethnicity, blood group, and phases of the menstrual cycle [41]. Other studies, such as the one reported by Miesbach et al. (2011), show that female carriers of hemophilia A can present with FVIII:C levels within the normal range but might also report a considerable tendency to bleed [42]. Even carriers with normal FVIII:C activity present an increased risk of bleeding as does this female patient. Incidence and intensity of bleeding symptoms of hemophilia A carriers are high and correlated with the phenotype of the male hemophilic relative and the underlying F8 gene mutation as in this case.

Patient Am3A has a severe HA diagnosis (F8 levels were at 1.3%) (Tables S1 and S2). This patient has variation c.789T > C that is altered within the used splice site, likely to disturb normal splicing, affecting protein features. Patients Am4A and Am7A both have severe phenotypes (Tables S1 and S2). Patient Am4A presents variation c.5506T > G and patient Am7A presents variation c.673A > G. Finally, patient Am5A has an unknown classification of his phenotype. His brothers have a confirmed diagnosis of HA (Tables S1 and S2); This patient presents variation c.6605A > T. This patient may have a higher risk of bleeding or a mild phenotype. For all cases, it is recommended to perform expression studies and functional analysis of the new variations found to demonstrate that these sequence variations affect F8 synthesis or function, as they are classified as variants with uncertain significance.

### 4.2. Genetic Analysis of F9 Gene

Patient Af1B is a possible carrier, without classification of disease severity (Table S3). The two variations found produce a protein strongly truncated with the loss of the peptidase S1 domain. This prevents the formation of a buried saline bridge [43]. Patient Am1B presents a severe HB phenotype with a family history of the disease in mother and siblings. The pathogenic variant rs137852261 found in this patient is associated with hereditary F9 deficiency (ID HGMD: CM940671). As reported by Ludwig et al. (1989) [44], a transition from C to T in the non-coding strand may explain these individual nucleotide substitutions. CpG dinucleotides are variation hot spots due to cytosine methylation followed by spontaneous deamination to thymine. No immune response related to the production of inhibitors has been reported in this patient as in the study [44,45]. Finally, in patient Af2B, a deletion of a single nucleotide was found. This deletion is predicted to produce a change in the reading frame or premature termination codons (PTCs), producing a strongly truncated protein, which may cause a “nonsense-mediated decay” (NMD). These results match the supporting criteria that include multiple bioinformatic predictors that support a detrimental effect and the patient’s phenotype, or family history is highly specific for a disease with a single genetic etiology.

The only high-frequency polymorphism in the *F9* is an A to G transition known as the Malmö G/A dimorphism mapped to exon 6 [8]. This single nucleotide polymorphism (ID rs6048) has a G allele frequency of 20%, however, none of our patients presented the polymorphism.

### 4.3. Genetic Analysis of VWF Gene

**Figure 9:**
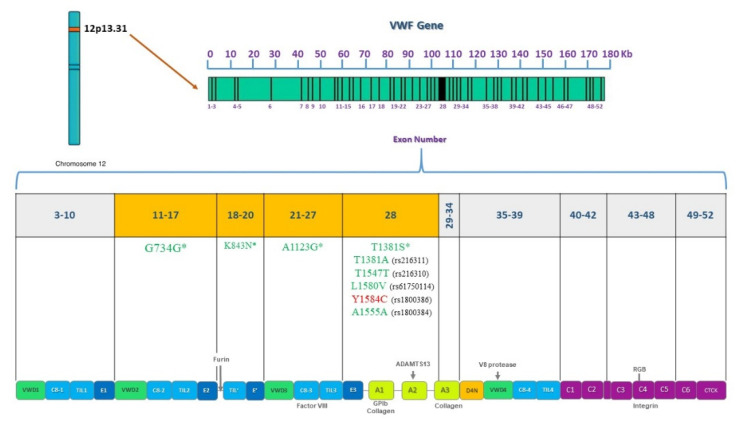
Pathogenic or benign variations found by *VWF* gene and protein domain. *VWF* is located on chromosome 12, 52 exons are shown and their corresponding domains in the VWF protein. Sequenced exons from 11 to 28 are highlighted in orange. Deleterious variations appear in red and benign variations in green. Asterisks indicate novel variations found.

For the VWD patient analysis, missense variations clustered in exon 28 usually associate with VWD types 2A, 2M, or 2B [21]. Patient Af1VW presents variations c.3835G > A and c.4133C > T. This patient is a compound heterozygote for defects in the *VWF* as found in the Eikenboom et al. study (1993) [46]. Since the variation is within the domains of the VWF that are involved in binding to the platelet membrane glycoprotein Ib as shown in Figure 9. The mutations may interfere with the ristocetin-induced VWF binding to platelets. This variation was found in patients with VWD type I diagnosis [46]. Variation c.4133C > T was found in a cohort study, where it was identified as type 1 VWD [47]. A broad range of different mutant *VWF* allele types were present in the study population and were inherited alone or in combination, resulting in a complex array of VWD types as in this case. Both variations correlate with low VWF levels (42.9) in this patient and these results coincide with the strong correlation criteria previously defined in the methodology section.

Patient Af2VW presents one non-synonymous substitution, Y1584C. This variation is a known disease variation (HGM CM031758). rs1800386 has been associated with the type 1 VWD phenotype as reported in previous studies [48,49,50,51]. As a future perspective, it is important to evaluate the multimer pattern to discard a type 2A VWD and confirm that the variation behaves as in the European and North American population as the cause of a type 1 VWD [48,49,50,51]. Patient Af3VW presents four variations in exons 19 and 25 (c.2479T > A, c.2482C > A, c.3291C > G, and c.3350T > G). It has been reported that variations in VWF that specifically decrease binding to F8 (type 2N VWD) map to both the TIL’ and E’ segments, suggesting a direct role in binding factor VIII [52]. Nevertheless, F8 levels in this patient are normal (Tables S4 and S5) so a VWD type 2N diagnosis must be carefully given. In VWD type 1, gene defects are spread throughout the entire *VWF* [53], so these variations found may be associated with a VWD type 1 phenotype.

For patient Am1VW, there was a clinical diagnosis of VWD type 2N (Tables S4 and S5). Variation c.2535C > A in exon 19 was found. The variation is associated with a loss of the VWF D3 domain (Figure 5). The variation found and a previous diagnosis of HA could correlate to a VWD type 2N diagnosis in this patient. Patient Af4VW presents the following variations: c.3252C > G, c.3297C > G, and c.3350T > G in exon 25 (Figure 5). It is important to mention that none of the VWD patients included in the study had been diagnosed with the multimer analysis, confirming the importance of complementing the clinical diagnosis with a genetic diagnosis. According to these protein effects, this patient might be a case of VWD type A2 with enhanced susceptibility to ADAMTS13-mediated proteolysis, impaired dimer and multimer assembly, loss of regulated storage, and intracellular retention. However, the different effects might also be associated with types 2N or 2M. A multimer analysis must be performed to fully diagnose this patient. For all cases, it is recommended to perform expression studies and functional analysis of the new variations found to demonstrate that these sequence variations affect VWF synthesis or function.

### 4.4. High-Resolution Melting Technique Validation

Two different patients with the same variation, c.440T > C, were tested and the same pattern of behavior for the samples in the different plot views is evidenced. Due to the position of the variation at the beginning of the amplicon, the signal magnitude was very close to that of the reference curve as shown in Figure 3B. However, the technique is sensitive enough to show differences. Nakagawa et al. (2016) [54] performed an HRM assay to detect a C to T transition located at nucleotide position c.1423 in exon 12 of the ADAMTS13 gene and demonstrated the potential benefit of the HRM technique for genotyping purposes. In replicate, the sensitivity and specificity of HRM were found to be identical to this study [54]. Better differentiation of the samples was evidenced when analyzing data with the programming code in Python vs. the graphs obtained with the commercial software.

According to [8,29,55], a mixture of male DNA with wildtype male DNA was necessary to generate an artificial heterozygote and detect the formed duplex. There is strong evidence of the benefit of mixing shown by our study, however, we also detected in the variation of the Am7A sample for exon 3 of *F8* that the difference plot revealed a greater difference of the sample without a DNA mixture with respect to normal samples than the artificial heterozygote (Figure S1). For this reason, we suggest including both types of samples in the exon analysis for males.

It is important to take into consideration that many authors have reported that a larger size of the amplicons is associated with lower efficiency of the method [8,11,12,13,54]. However, confirmed variations present in exons 8.1 and 8.3 of the *F9* from Af2B, Am1B, and Af1B patients, and variation on exon 1 of the *F8*, were detected despite the size of the amplicon (800 bp). In these cases, domain analysis allowed us to improve the previously reported sensitivity of 93% [11] to 95% at a lower cost. Unlike the methodology used by other studies where 52 reactions per patient were needed to carry out the gene analysis [10,11], we only used 35 reactions per patient. We propose a protocol for molecular analysis using HRM that supports the clinical diagnosis of patients with hemophilia, shown in Figure 7.

## 5. Conclusions

Twenty-seven novel variations were found in this study. As shown in this study, sequencing of exons associated with VWD type 2 phenotypes helps to better guide VWD patient diagnosis and should be incorporated into the routine analysis.

HRM is a promising tool for the molecular diagnosis of these coagulopathies as it has proven to be a sensitive technique that can be used as a diagnostic method and should be incorporated in developing countries. Given the great socioeconomic impact of these diseases on different countries’ health systems, HRM represents significant cost differences in contrast to direct gene sequencing that can be translated not only into a greater number of patients who can access an accurate diagnosis but also into an effective treatment and a better quality of life.

The use of programming code improves the objectivity and the performance of the measurements generated with the HRM analysis. Analysis discriminated by sex improves the sensitivity and, in the case of amplicons with a length greater than 300 bp, domain analysis also improves sensitivity, making the analysis more cost-effective. Among the advantages, we include the objective identification of the Tm1 and Tm2 since these temperatures represent subjective parameters when working only with the LightCycler program. Open-source codes allow continuous improvement of the analysis by applying more functions that allow a more objective analysis that is not possible in commercial programs.

## Figures and Tables

**Table 1:** Primer sequences used in PCR amplifications of the F9 and VWF genes with the different PCR conditions for each primer pair.

Primer	Sequence	PCR Conditions *
VWFE17F VWFE17R	CGATATTGAAAGGCCAGGCAG CCCAGAAATGAAGGCGATCCT	94 °C for 30 s, 56 °C for 45 s, and 72 °C for 90 s.
VWFE18F VWFE18R	CACAGACTCTAGGGGACCAA GCCTACAAGAAAACTGAAGGGC	94 °C for 30 s, 51 °C for 45 s, and 72 °C for 30 s.
VWFE19F VWFE19R	GAACCCATGTTTGCCAAGCC CTATGCAGGATGGACACAGGT	94 °C for 30 s, 51 °C for 45 s, and 72 °C for 90 s.
VWFE20F VWFE20R	GCTACAGGTCCTCAACTTCCT GAAACCAGACCCCAGAGTTGT	94 °C for 30 s, 51 °C for 45 s, and 72 °C for 45 s.
VWFE21F VWFE21R	ACACACCTGAGGGCATTCAC TCCTCTTTAATGGCTGTGCGT	94 °C for 30 s, 58 °C for 45 s, and 72 °C for 45 s.
VWFE22F VWFE22R	GCCCTTCATCCTCCTGGATTTC GTCCCCAACAAGATGAAGCA	94 °C for 30 s, 51 °C for 45 s, and 72 °C for 30 s.
VWFE23/24F VWFE23/24R	AAGGAAGCTCAGGAATGGGT ACTCTGTGTCCATACCACCA	94 °C for 30 s, 55 °C for 45 s, and 72 °C for 90 s.
VWFE25F VWFE25R	GCCAGCAGCACTGCATTATT CTTGGCCATCCAGTCCCTAC	94 °C for 30 s, 51 °C for 45 s, and 72 °C for 45 s.
VWFE28-1F VWFE28-1R	GCTCAGAAGTGTCCACAGGTT GACGAACGCCACATCCAGAA	94 °C for 30 s, 57 °C for 45 s, and 72 °C for 90 s.
VWFE28-2F VWFE28-2R	TCAAGCAGATCCGCCTCATC GATGCATGTAGCACCAAGGC	94 °C for 30 s, 55 °C for 45 s, and 72 °C for 90 s.
FIXE1 + promoterF FIXE1 + promoterR	CCCATTCTCTTCACTTGTCC CCTAGCTAACAAAGAACCAGT	94 °C for 30 s, 50 °C for 45 s, and 72 °C for 90 s.
FIXE2-3F FIXE2-3R	AGAGATGTAAAATTTTCATGATGTT GCAGAGAAAAAACCCACATAAT	95 °C for 30 s, 50 °C for 90 s, and 72 °C for 90 s.
FIXE4F FIXE4R	GCTGGCTTCCAGGTCAGTAG CCAGTTTCAACTTGTTTCAGAGGG	95 °C for 30 s, 51 °C for 30 s, and 72 °C for 90 s.
FIXE5F FIXE5R	CATGAGTCAGTAGTTCCATGTACTTT TGTAGGTTTGTTAAAATGCTGAAGTT	95 °C for 30 s, 60 °C for 30 s, and 72 °C for 90 s.
FIXE6F FIXE6R	GTGACAAGGATGGGCCTCAA TGGTTAGTGCTGAAACTTGCC	95 °C for 30 s, 60 °C for 30 s, and 72 °C for 90 s.
FIXE7F FIXE7R	ATTTTGTTTTCACAGGTTGTTTTGA TATTCTTTCTGTGTATTTACCTGCG	95 °C for 30 s, 58 °C for 45 s, and 72 °C for 90 s.
FIXE8-1F ** FIXE8-1R	AGGTCAGTGGTCCCAAGTAGT TTCCTCTAGTGGTTCCATTTTCT	95 °C for 30 s, 50 °C for 90 s, and 72 °C for 90 s.
FIXE8-2F ** FIXE8-2R	TGAAAATTAACAGGGCCTCTCAC AGCTCTTAGAATGGCTTATTGCT	95 °C for 30 s, 54 °C for 90 s, and 72 °C por 90 s.
FIXE8-3F ** FIXE8-3R	CTATCAAACCCAGACTTGCTTCC TTTTGTCAGTAGTCCCATGCATCA	95 °C for 30 s, 56 °C for 45 s, and 72 °C for 90 s.

* Samples were amplified under the same following PCR conditions: 95 °C for 2 min, followed by 30 cycles that vary according to each primer pair and a final extension of 72 °C for 5 min. ** Given the size of exon 8 (1935 bp) it was divided into three overlapping segments: 8-1, 8-2, and 8-3.

**Table 2:** Genotype results of patients with HA, HB, or VWD diagnosis.

Code	Gender	Diagnosis	Genetic Variant *	Exon **	Genotype	Amino Acid Substitution	Prediction Software Analysis	dbSNP ID/HGMD ***
Af1a	Female	Hemophilia A FVIII (%): 24.3 Coagulometric Technique Reference Values 50–150% Referenced her brother’s Values at 1.2%	c.3690_3691insG	14D	Heterozygous	p.(Pro1231*)	Mutation Taster: Disease Causing Prob: 1	Novel
			c.5440G > A	16	Heterozygous	p.(Asp1814Asn)	Sift: Damaging Score: 0.0	Novel
							PhD-SNP: Disease	
							Polyphen: Benign Score: 0.014	
							Mutation Taster: Polymorphism Prob: 0.999999996251468	
							Ensembl Impact: Moderate	
			c.6605A > T	24	Heterozygous	p.(Lys2202Ile)	Sift: Damaging Score: 0.02	Novel
							PhD-SNP: Disease	
							Polyphen: Possibly Damaging Score: 0.693	
							Mutation Taster: Polymorphism Prob: 0.9999999407418885	
							Ensembl Impact: Modifier	
Af2a	Female	Hemophilia A FVIII (%): 71.3 Coagulometric Technique Reference Values 50–150%	c.673A > G	6	Homozygous	p.(Lys225Glu)	PhD-SNP: Disease	Novel
							Polyphen: Possibly Damaging Score: 0.657	
							Mutation Taster: Disease Causing Prob: 0.999729299879278	
							Ensembl Impact: Modifier	
			c.6605A > T	24	Heterozygous	p.(Lys2202Ile)	Sift: Damaging Score: 0.02	Novel
							PhD-SNP: Disease	
							Polyphen: Possibly Damaging Score: 0.693	
							Mutation Taster: Polymorphism Prob: 0.9999999407418885	
							Ensembl Impact: Modifier	
Am1A	Male	Hemophilia A FVIII (%): 4 Coagulometric Technique Reference Values 50–150%	c.5375T > A	16	Hemizygous	p.(Val1792Glu)	Sift: Damaging Score: 0	Novel
							PhD-SNP: Disease	
							Polyphen: Probably Damaging Score: 1.000	
							Mutation Taster: Disease Causing Prob: 0.999999863121858	
							Ensembl Impact: Modifier	
Am2A	Male	Hemophilia A FVIII (%): 0.9 Coagulometric Technique Reference Values 50–150%	c.203C > T	2	Hemizygous	p.(Thr68Ile)	Sift: Tolerated Score: 0.46	CM1212705
							PhD-SNP: Neutral	
							Polyphen: Probably Damaging Score: 0.94	
							Mutation Taster: Disease Causing Prob: 0.545256099530899	
							Ensembl Impact: Moderate	
Af3A	Female	Hemophilia A FVIII (%): 30.0 Coagulometric Technique Reference Values 50–150% Referenced Her Son’s Values at 0.33%	c.440T > C	4	Homozygous	p.(Val147Ala)	Sift: Damaging Score: 0	Novel
							PhD-SNP: Disease	
							Polyphen: Probably Damaging	
							Score: 0.973	
							Mutation Taster: Disease Causing Prob: 0.999979328541858	
Am3A	Male	Hemophilia A FVIII (%): 1.3 Coagulometric Technique Reference Values 50–150%	c.789T > C	7	Hemizygous	p.(Gly263Gly)	PhD-SNP: Disease	Novel
							Mutation Taster: Disease Causing Prob: 0.999999999999972	
							Ensembl Impact: Modifier	
Af4A	Female	Hemophilia A FVIII (%): 83.2 Coagulometric Technique Reference Values 50–150%	c.440T > C	4	Heterozygous	p.(Val147Ala)	Sift: Damaging Score: 0	Novel
							PhD-SNP: Disease	
							Polyphen: Probably Damaging	
							Score: 0.973	
							Mutation Taster: Disease Causing Prob: 0.999979328541858	
			c.5440G > A	16	Homozygous	p.(Asp1814Asn)	Sift: Damaging Score: 0.02	Novel
							PhD-SNP: Disease	
							Polyphen: Benign Score: 0.014	
							Mutation Taster: Polymorphism Prob: 0.999999996251468	
							Ensembl Impact: Moderate	
Am4A	Male	Hemophilia A FVIII (%): 0.4 Coagulometric Technique Reference Values 50–150%	g.4266C > T	1	Hemizygous	5’UTR variant	Mutation Taster: Polymorphism Prob: 0.999997026960022	Novel
							Ensembl: Modifier	
			c.5506T > G	16	Hemizygous	p.(Trp1836Gly)	Sift: Damaging Score: 0	Novel
							PhD-SNP: Disease	
							Polyphen: Probably Damaging Score: 1	
							Mutation Taster: Disease Causing Prob: 0.999999201432753	
Am5A	Male	Hemophilia A FVIII (%): 45.7 Coagulometric Technique Reference Values 50–150%	c.5535T > A	16	Hemizygous	p.(Thr1845Thr)	PhD-SNP: Neutral	Novel
							Mutation taster: Polymorphism Prob: 0.99999573313174	
							Ensembl Impact: Modifier	
			c.6605A > T	24	Hemizygous	p.(Lys2202Ile)	Sift: Damaging Score: 0.02	Novel
							PhD-SNP: Disease	
							Polyphen: Possibly Damaging Score: 0.781	
							Mutation Taster: Polymorphism Prob: 0.999999940741885	
							Ensembl Impact: Moderate	
Am6A	Male	Hemophilia A FVIII (%): 14 Coagulometric Technique Reference Values 50–150%	c.673A > G	6	Hemizygous	p.(Lys225Glu)	Sift: Tolerated Score: 0.12	Novel
							PhD-SNP: Disease	
							Polyphen: Possibly Damaging Score: 0.657	
							Mutation Taster: Disease Causing Prob: 0.999729299879278	
							Ensembl Impact: Moderate	
			c.6605A > T	24	Hemizygous	p.(Lys2202Ile)	Sift: Damaging Score: 0.02	Novel
							PhD-SNP: Disease	
							Polyphen: Possibly Damaging Score: 0.781	
							Mutation Taster: Polymorphism Prob: 0.999999940741885	
							Ensembl Impact: Moderate	
Am7A	Male	Hemophilia A FVIII (%): 0.83 Coagulometric Technique Reference Values 50–150%	c.673A > G	6	Hemizygous	p.(Lys225Glu)	Sift: Tolerated Score: 0.12	Novel
							PhD-SNP: Disease	
							Polyphen: Possibly Damaging Score: 0.657	
							Mutation Taster: Disease Causing Prob: 0.999729299879278	
							Ensembl Impact: Moderate	
Am1B	Male	Hemophilia B FIX (%): 0.1 Coagulometric Technique Reference Values 50–150%	c.1150C > T	8.1	Hemizygous	p.Arg384 *	Mutation Taster: Disease Causing Model: complex_aae, prob: 1	rs137852261 CM940671
							Ensembl Impact: Modifier	
Af1B	Female	Hemophilia B FIX (%): Unknown Coagulometric Technique Reference Values 50–150% Referenced Her Son’s Values at 1.5%	c.810_811insG	7	Heterozygous	p.(Thr271Dfs*4)	Mutation Taster: Disease Causing Model: complex_aae, prob: 1	Novel
							Ensembl impact: Modifier	
			g.32185T > C	8.3	Heterozygous	Alteration region: 3’UTR	Mutation Taster: Polymorphism Prob: 0,999989953358595	Novel
Af2B	Female	Hemophilia B FIX (%): Unknown Coagulometric Technique Reference Values 50–150% Referenced Her Son’s Values at 0.4%	c.1038_1038delC	8.1	Homozygous	p. (Lys347Nfs *)	Mutation Taster: Disease Causing Model: complex_aae, prob: 1	Novel
							Ensembl Impact: Modifier	
Af1VW	Female	von Willebrand Disease von Willebrand Factor (Antigen): 42.9 Technique: Immunocapture Reference Values 50–160%	c.2454G > T	19	Heterozygous	p. (Glu818Asp)	Sift: Tolerated Score: 0.21	Novel
							PhD-SNP: Neutral	
							Polyphen: Benign Score: 0.006	
							Mutation Taster: Polymorphism Prob: 0.99792257832411	
			c.2529G > C	19	Homozygous	p.(Lys843Asn)	Sift: Tolerated Score: 0.11	Novel
							PhD-SNP: Neutral	
							Polyphen: Benign Score: 0.153	
							Mutation Taster: Polymorphism Prob: 0.997287667014106	
			c.3835G > A	28A	Heterozygous	p.Val1279Ile	Sift: Damaging Score: 0.04	rs61749376 CM931400
							PhD-SNP: Neutral	
							Polyphen: Possibly damaging	
							Score: 0.735	
							Mutation Taster: Disease causing Prob: 0.99844456886186	
			c.4027A > G	28A	Heterozygous	p.Ile1343Val	Sift: Tolerated Score: 0.07	rs150923481
							PhD-SNP: Neutral	
							Polyphen: Benign Score: 0.087	
							Mutation Taster: Polymorphism Prob: 0.982659315673669	
			c.4133C > T	28A	Heterozygous	p.Ser1378Phe	Sift: Damaging Score: 0.01	rs61750073 CM070351
							PhD-SNP: Disease	
							Polyphen: Probably damaging	
							Score: 1.000	
							Mutation Taster: Polymorphism Prob: 0.999732762130257	
			c.4141A > G	28A	Homozygous	p.Thr1381Ala	Sift: Tolerated Score: 1	rs216311
							PhD-SNP: Neutral	
							Polyphen: Benign Score: 0.000	
							Mutation Taster: Polymorphism Prob: 0.999999999999837	
			c.4641T > C	28B	Homozygous	p.Thr1547Thr	Sift: Tolerated Score: 1	rs216310
							PhD-SNP: Neutral	
							Mutation Taster: Polymorphism Prob: 0.999466147207589	
			c.4738C > G	28B	Heterozygous	p. Leu1580Val	Sift: Tolerated Score: 0.56	rs61750114 CM095110
							PhD-SNP: Neutral	
							Polyphen: Benign Score: 0.197	
							Mutation Taster: Polymorphism Prob: 0.99999993020585	
Af2VW	Female	von Willebrand Disease von Willebrand Factor (Antigen): 46.2 Technique: Immunocapture Reference Values 50–160%	c.3368C > G	25	Heterozygous	p.(Ala1123Gly)	Sift: Damaging Score: 0.05	Novel
							PhD-SNP: Neutral	
							Polyphen: Benign Score: 0.024	
							Mutation Taster: Polymorphism Prob: 0.99999999966864	
			c.4141A > T	28A	Heterozygous	p.(Thr1381Ser)	Sift: Damaging Score: 0.01	Novel
							PhD-SNP: Neutral	
							Polyphen: Benign Score: 0.030	
							Mutation Taster: Polymorphism Prob: 0.999999999999947	
			c.4751A > G	28B	Heterozygous	p.Tyr1584Cys	Sift: Damaging Score: 0	rs1800386 CM031758
							PhD-SNP: Disease	
							Polyphen: Probably damaging Score: 0.987	
							Mutation Taster: Polymorphism Prob: 0.999999120870778	
Af3VW	Female	von Willebrand Disease von Willebrand Factor (Antigen): 34.5 Technique: Immunocapture Reference Values 50–160%	c.2479T > A	19	Heterozygous	p.(Cys827Ser)	Sift: Damaging Score: 0	Novel
							PhD-SNP: Disease	
							Polyphen: Possibly Damaging Score: 1.000	
							Mutation Taster: Disease causing Prob: 0.999999938099734	
			c.2482C > A	19	Heterozygous	p.(Pro828Thr)	Sift: Damaging Score: 0	Novel
							PhD-SNP: Neutral	
							Polyphen: Possibly Damaging Score: 1.000	
							Mutation Taster: Disease Causing Prob: 0.999999953987469	
			c.2529G > C	19	Heterozygous	p.(Lys843Asn)	Sift: Tolerated Score: 0.11	Novel
							PhD-SNP: Neutral	
							Polyphen: Benign Score: 0.153	
							Mutation Taster: Polymorphism Prob: 0.997287667014106	
			c.2770C > A	21	Heterozygous	p.(Arg924Arg)	Sift: Tolerated Score: 1	Novel
							PhD-SNP: Neutral	
							Mutation Taster: Disease Causing Prob: 0.999999860094315	
			c.3291C > G	25	Heterozygous	p.(Cys1097Trp)	Sift: Damaging Score: 0	Novel
							PhD-SNP: Neutral	
							Polyphen: Possibly Damaging Score. 0.641	
							Mutation Taster: Disease Causing Prob: 0.999997683583057	
			c.3350T > G	25	Heterozygous	p.(Val1117Gly)	Sift: Damaging Score: 0	Novel
							PhD-SNP: Disease	
							Polyphen: Possibly Damaging Score. 0.642	
							Mutation Taster: Disease Causing Prob: 0.881353491915509	
			c.3951C > T	28A	Heterozygous	p.Ala1317Ala	Sift: Tolerated Score: 1	rs561155315
							PhD-SNP: Neutral	
							Mutation Taster: Disease Causing Prob: 0.999999999983201	
			c.4141A > G	28A	Homozygous	p.Thr1381Ala	Sift: Tolerated Score: 1	rs216311
							PhD-SNP: Neutral	
							Polyphen: Benign Score: 0.000	
							Mutation Taster: Polymorphism Prob: 0.999999999999837	
			c.4641T > C	28B	Homozygous	p.Thr1547Thr	Sift: Tolerated Score: 1	rs216310
							PhD-SNP: Neutral	
							Mutation Taster: Polymorphism Prob: 0.999466147207589	
			c.4738C > G	28B	Heterozygous	p.Leu1580Val	Sift: Tolerated Score: 0.59	rs61750114
							PhD-SNP: Neutral	
							Polyphen: Benign Score: 0.197	
							Mutation Taster: Polymorphism Prob: 0.99999993020585	
Am1VW	Male	von Willebrand Disease von Willebrand Factor (Antigen): 56 Technique: Immunocapture Reference Values 50–160%	c.2535C > A	19	Homozygous	p.(Gly845Gly)	Sift: Tolerated Score: 0.52	Novel
							PhD-SNP: Neutral	
							Mutation Taster: Disease Causing Prob: 1	
			c.4141A > G	28A	Homozygous	p.Thr1381Ala	Sift: Tolerated Score: 1	rs216311
							PhD-SNP: Neutral	
							Polyphen: Benign Score: 0.000	
							Mutation Taster: Polymorphism Prob: 0.999999999999837	
			c.4641T > C	28B	Homozygous	p.Thr1547Thr	Sift: Tolerated Score: 1	rs216310
							PhD-SNP: Neutral	
							Mutation Taster: Polymorphism Prob: 0.999466147207589	
Af4VW	Female	von Willebrand Disease von Willebrand Factor (Antigen): Unknown Technique: Immunocapture Reference Values 50–160%	c.3252C > G	25	Heterozygous	p.(Cys1084Trp)	Sift: Damaging Score: 0	Novel
							PhD-SNP: Disease	
							Polyphen: Possibly Damaging Score: 1.000	
							Mutation Taster: Disease Causing Prob: 0.999999999989141	
			c.3297C > G	25	Heterozygous	p.(Cys1099Trp)	Sift: Damaging Score: 0	Novel
							PhD-SNP: Disease	
							Polyphen: Possibly Damaging Score: 1.000	
							Mutation Taster: Disease Causing Prob: 0.999999999413986	
			c.3350T > G	25	Heterozygous	p.(Val1117Gly)	Sift: Damaging Score: 0	Novel
							PhD-SNP: Disease	
							Polyphen: Possibly Damaging Score: 0.642	
							Mutation Taster: Disease Causing Prob: 0.881353491915509	
			c.4141A > G	28A	Homozygous	p.Thr1381A	Sift: Tolerated Score: 1	rs216311
							PhD-SNP: Neutral	
							Polyphen: Benign Score: 0.000	
							Mutation Taster: Polymorphism Prob: 0.999999999999837	
			c.4641T > C	28B	Homozygous	p.Thr1547Thr	Sift: Tolerated Score: 1	rs216310
							PhD-SNP: Neutral	
							Mutation Taster: Polymorphism Prob: 0.999466147207589	

* The mutation numbering was according to the complete mRNA sequence to keep consistency with other published results; the amino acid numbering is given for the mature processed protein. F8: ENST00000360256; F9: ENST00000218099; VWF: ENST00000261405. ** F8 exons 14 and 26, F9 exon 8, and VWF exon 28 were divided into different PCR reactions for sequencing. *** HGMD: corresponds to the Human Gene Variation Database used by Mutation Taster to search for reported variations. Factor level measurements shown in the table are only one measurement in time that corresponds to the last measurement taken in the medical history of the patients included in the study. The patients included in the study come from unrelated families, therefore the codes do not reflect any type of relationship between the patients.

**Table 3:** Classification of variations in patients with HA, HB, or VWD according to the clinical significance.

*F8* Variations	SNP Position	Variation Effect	Reported Variations (Human Gene Variation Database ID or rs ID)	Total: 11
Variant with uncertain significance	c.3690_3691insG	p.(Pro1231 *)	novel	11
	c.5440G > A	p.(Asp1814Asn)	novel	
	c.6605A > T	p.(Lys2202Ile)	novel	
	c.673A > G	p.(Lys225Glu)	novel	
	c.5375T > A	p.(Val1792Glu)	novel	
	c.203C > T	p.(Thr68Ile)	CM1212705	
	c.440T > C	p.Val147Ala	novel	
	c.789T > C	p.(Gly263Gly)	novel	
	c.5506T > G	p.(Trp1836Gly)	novel	
	c.5535T > A	p.(Thr1845Thr)	novel	
	g.4266C > T	5’UTR variant	novel	
***F9*** **variations (HB)**	**SNP position**	**Variation effect**	**Reported Variations**	**Total: 4**
Pathogenic variant	c.1150C > T	p.Arg384 *	rs137852261/CM940671	1
Variant with uncertain significance	c.810_811insG	p.(Thr271Dfs*4)	novel	3
	g.32185T > C	3’UTR variant	novel	
	c.1038_1038delC	p. (Lys347Nfs *)	novel	
***VWF* variations (VWD)**	**SNP position**	**Variation effect**	**Reported Variations**	**Total: 19**
Likely pathogenic variant	c.4751A > G	p.Tyr1584Cys	rs1800386/CM031758	1
Likely benign variant	c.4141A > G	p.Thr1381Ala	rs216311	2
	c.4641T > C	p.Thr1547Thr	rs216310	
Variant with uncertain significance	c.4027A > G	p.Ile1343Val	rs150923481	17
	c.4133C > T	p.Ser1378Phe	rs61750073/CM070351	
	c.4738C > G	p.Leu1580Val	rs61750114/CM095110	
	c.3951C > T	p.Ala1317Ala	rs561155315	
	c.2454G > T	p. (Glu818Asp)	novel	
	c.2529G > C	p.(Lys843Asn)	novel	
	c.3835G > A	p.Val1279Ile	rs61749376 CM931400	
	c.3368C > G	p.(Ala1123Gly)	novel	
	c.2479T > A	p.(Cys827Ser)	novel	
	c.2482C > A	p.(Pro828Thr)	novel	
	c.2770C > A	p.(Arg924Arg)	novel	
	c.3291C > G	p.(Cys1097Trp)	novel	
	c.2535C > A	p.(Gly845Gly)	novel	
	c.3252C > G	p.(Cys1084Trp)	novel	
	c.3350T > G	p.(Val1117Gly)	novel	
	c.3297C > G	p.(Cys1099Trp)	novel	

“*” is used to describe a stop Codon according to HGVS Recommendations for the Description of Sequence Variants.

**Table 4:** HRM analysis results for exons of the *F8* and *F9* genes.

Exon	Patient	Patient Diagnosis	Variation	HRM Validation *
1	Am4a	HA	g.4266C > T	V
2	Am2a	HA	c.203C > T	V
4	Af3a	HA	c.440T > C	V
4	Af4a	HA	c.440T > C	V
6	Af2a	HA	c.673A > G	N
6	Am6a	HA	c.673A > G	V
6	Am7a	HA	c.673A > G	V
7	Am3a	HA	c.789T > C	V
16	Af1a	HA	c.5440G > A	V
16	Af4a	HA	c.5440G > A	V
16	Am1a	HA	c.5375T > A	V
16	Am4a	HA	c.5506T > G	V
16	Am5a	HA	c.5535T > A	V
24	Af1a	HA	c.6605A > T	V
24	Af2a	HA	c.6605A > T	V
24	Am5a	HA	c.6605A > T	V
24	Am6a	HA	c.6605A > T	V
7	Af1b	HB	c.810_811insG	V
8.1	Af2b	HB	c.1038_1038delC	V
8.1	Am1b	HB	c.1150C > T	V
8.3	Af1b	HB	g.32185T > C	V

* V: validated, N: not validated. Total: V: 20/21; N: 1/21.

## Data Availability

Data are contained within the article or Supplementary Material. The data presented in this study are available at https://gitfront.io/r/user-5547184/d3ec1062bc7d0d2e3039b74770330c06f510d65e/hrm-analysis/, accessed on 20 August 2021.

## Supplementary Materials

The following are available online at https://www.mdpi.com/article/10.3390/genes12111807/s1, Figure S1: Variation c.673A > G HRM analysis, with male discrimination. Difference plot shows greater difference of the sample without DNA mixture (shown with a red arrow) with respect to normal samples than the artificial heterozygote; Figure S2: Variation c.6605A > T HRM analysis, with female discrimination; Figure S3: *F8* exon 24 HRM analysis, with male discrimination; Table S1: Phenotype characteristics of hemophilia A patients; Table S2: Phenotype characteristics of hemophilia A patients; Table S3: Phenotype characteristics of hemophilia B patients; Table S4: Phenotype characteristics of von Willebrand Disease patients; Table S5: Phenotype characteristics of von Willebrand Disease patients.
