# Supplementary material for: Genetic and Bioinformatic Strategies to Improve Diagnosis in Three Inherited Bleeding Disorders in Bogotá, Colombia
Source: Genes (Basel). 2021 Nov 18;12(11):1807. doi: 10.3390/genes12111807 (PMC8625804; doi:10.3390/genes12111807)
Supplement: Supplementary file 1 [file genes-12-01807-s001.zip › Table S1.pdf]

Table S1. Phenotype characteristics of Hemophilia A patients.

Patient code	Sex	Age	Blood type	Rh	Race	Diagnosis	First consultation reason	Family background	Genetic Counseling	Disease Classification	FVIII (%) coagulometric method reference values 50-150%[1]	von Willebrand Factor (Antigen) technique: immunocapture reference values 50-160% [2.]
Af1A	Female	25	O	+	Caucasian	carrier	Family history	mother and brothers	no	carrier	24,3	60,0
Brother of Af1A*	Male	31	A	+	Caucasian	hemophilia A	Family history	mother and brothers	no	moderate with severe presentation pattern	1,2	NA
Af2A	Female	29	O	+	Caucasian	carrier	Family history	mother brothers. 18-month-old brother deceased. Dosage of coagulation FVIII evaluated at 1%.	no	carrier	71,3	75,0
Am1A	Male	44	O	+	Caucasian	hemophilia A	Hemorrhages	Brothers and cousins	no	mild	4	70
Am2A	Male	59	O	+	Caucasian	hemophilia A	Hemorrhages	Nephew and Cousins	no	severe	0,9	NA
Af3A	Female	36	B	+	Caucasian	carrier	Abundant menorragia – Son with hemophilia A diagnosis	no family background	no	carrier	108.4 (last data at the time of entering the study) 30.0 (data considered at the time of diagnosis)	116,6

Son of Af3A*	Male	3	O	+	Caucasian	hemophilia A	bruises	no family background	no	severe	0,33	NA
Am3A	Male	28	O	+	Native	hemophilia A	bruises, mucous bleeding	mother father and brothers	no	severe	1,3	NA
Af4A	Female	68	O	+	Caucasian	carrier	family history-Hemorrhages	mother, brothers, cousins and grandfather	no	carrier	83,2	103,0
Am4A	Male	40	A	+	Caucasian	hemophilia A	Hemorrhages	no family background	yes	severe	0,4	NA
Am5A	Male	61	A	+	Caucasian	hemophilia A	Family history	Brothers and cousins	no	mild	45,7	NA
Am6A	Male	83	O	-	Caucasian	hemophilia A	coronary bypass surgery	Nephew	no	mild	14	NA
Am7A	Male	27	O	+	Native	hemophilia A	Hemorrhages	Cousin	no	severe	0,83	NA

**Patients not included in the study. Blood samples from their relatives with a carrier status were taken.NA: No answer.*
Factor level measurements shown in the table are only one measurement in time that corresponds to the last measurement taken in the medical history of the patients included in the study. Some factor levels can be altered by pregnancy, age, hormonal changes, among others.

[1:] **COAGULATION FACTOR VIII (Antihemophilic Factor A):** Sample conditions included: Platelet-poor recentrifuged citrated plasma. 1 mL of plasma was separated and frozen immediately in a plastic tube. Sample, free of hemolysis and lipemia, was ship frozen on dry ice. The method used was the clot formation with a reference interval of 50 - 150%. Specifications of the assay: One-stage assay. Clot of an atapa, automated performed on a CS2100i coagulometer. Reagents: Calibrator plasma, normal control plasma, abnormal control plasma, FVIII deficient plasma, PTT reagent: Actin FSL, Calcium chloride, owren buffer.

[2:] **VON WILLEBRAND ANTIGEN FACTOR (VWF:Ag):** Sample conditions included: Platelet-poor recentrifuged citrated plasma. 1 mL of plasma was separated and frozen immediately in a plastic tube. Sample, free of hemolysis and lipemia, was ship frozen on dry ice. The method used was Immunoturbidimetry, automated performed on a CS2100i coagulometer, with a reference interval of 50-160%. Reagents: Calibrator plasma, normal control plasma, abnormal control plasma, Von Willebrand Antigen measurement kit. Specifications of the assay: Haberichter, S. 2016. Von Willebrand factor propeptide: biology and clinical utility. Blood Journal. 126 (15). 1753-1761
