# Supplementary material for: Genetic and Bioinformatic Strategies to Improve Diagnosis in Three Inherited Bleeding Disorders in Bogotá, Colombia
Source: Genes (Basel). 2021 Nov 18;12(11):1807. doi: 10.3390/genes12111807 (PMC8625804; doi:10.3390/genes12111807)
Supplement: Supplementary file 1 [file genes-12-01807-s001.zip › Table S2.pdf]

Table S2. Phenotype characteristics of Hemophilia A patients.

Patient code	Ristocetin cofactor (%) technique: Aggregometry, Immunoturbidimetry with a reference interval of Blood Group O: 53.0 - 152.8 and Blood Groups A, B, AB: 71.2 - 178.5[1]	Multimer analysis	RIPA (%) technique: aggregometry reference values: Ristocetin 1.2: 50 - 100% Ristocetin 0.5: no aggregation observed[2]	Scheme of treatment	Administration	Factor type	Dose (UI)	Age of treatment start	Medicine brands	Inhibitor test result	Episodes of bleeding in the last year	Hemarthrosis	Occurrences of Hemarthrosis	Chronic Hemophilic Arthropathy
Af1a	59,0	NA	83,4	No treatment	NA	NA	NA	NA	NA	-	0	no	0	no
Brother of Af1A*	NA	NA	NA	Prophylaxis	3 times/week	Plasma derivative	2000	27	Hemofil: Plasma derivative	-	0	yes (Hemophilic arthropathy of right elbow, knees, ankles and left shoulder.)	4	yes
Af2A	NA	NA	NA	No treatment	NA	NA	NA	NA	NA	-	1	no	0	no
Am1A	NA	NA	NA	on demand	4 times in total	Recombinant	NA	1	NA	-	365 daily rectal bleeding	no	0	no
Am2A	NA	NA	NA	Prophylaxis	3 times/week	Recombinant	2000	48	Advate-octanate-beriate-xyntha	+	4	yes	4	yes
Af3A	120,9	Multimers present in normal amounts	NA	No treatment	NA	NA	NA	NA	NA	-	NA	NA	0	no
Son of Af3A*	NA	NA	NA	Prophylaxis	2 times/week	Recombinant	5	2	ADVATE-Xyntha.	-	0	yes	1	no
Am3A	NA	NA	NA	on demand	5 times in total	Recombinant	2500	17	Advate-Xyntha	-	NA	no	0	no

Af4A	NA	NA	NA	No treatment	NA	NA	NA	NA	NA	NA	NA	NA	0	NA
Am4A	NA	NA	NA	Prophylaxis	3 times/week	Plasma derivative	1500	20	Haemoctin	-	4	yes	4	yes
Am5A	NA	NA	NA	No treatment	NA	NA	NA	NA	NA	-	NA	NA	0	NA
Am6A	NA	NA	NA	on demand	3 times in total	Recombinant	2000	64	Xyntha	-	no	no	no	no
Am7A	NA	NA	NA	Prophylaxis	3 times/week	Plasma derivative	2000	11	Hemofil: Plasma derivative	+	no	no	no	yes

**Patients not included in the study. Blood samples from their relatives with a carrier status were taken.*

NA: No answer.

[1:] **FUNCTIONAL VON WILLEBRAND FACTOR: Activity (VWF: Ac), Ristocetin Cofactor (VWF: Rco):** Sample conditions included: Platelet-poor recentrifuged citrated plasma. 1 mL of plasma was separated and frozen immediately in a plastic tube. Sample, free of hemolysis and lipemia, was ship frozen on dry ice. The method used was Aggram of Helena Laboratories with a reference interval of Blood Group O: 53.0 - 152.8 and Blood Groups A, B, AB: 71.2 - 178.5. Reagents: Calibrator plasma, normal control plasma, abnormal control plasma, Ristocetin, normal platelets fixed in formalin, owren buffer. Specifications of the assay: Federici, A.B. 2016. Current and emerging approaches for assessing von Willebrand disease in 2016. International Journal of Laboratory Hematology.38 (1). 41-49

[2:] **PLATELET AGGREGATION RIPA 2 DILUTIONS: RISTOCETIN 1.2 and 0.5:** Sample conditions included: Citrated whole blood. 5 mL at room temperature in a plastic tube. Sample, free of hemolysis and lipemia. The method used was Turbidimetry with a reference interval: Ristocetin 1.2: 50 - 100% Ristocetin 0.5: no aggregation observed. Specifications of the assay: Hayward, C., Moffat, M., Graf, L. 2014. Technological advances in diagnostic testing for von Willebrand disease: new approaches and challenges. International Journal of Laboratory Hematology. 36, 334-340
