# Supplementary material for: Genetic and Bioinformatic Strategies to Improve Diagnosis in Three Inherited Bleeding Disorders in Bogotá, Colombia
Source: Genes (Basel). 2021 Nov 18;12(11):1807. doi: 10.3390/genes12111807 (PMC8625804; doi:10.3390/genes12111807)
Supplement: Supplementary file 1 [file genes-12-01807-s001.zip › Table S3.pdf]

Table S3. Phenotype characteristics of Hemophilia B patients.

Patient code	Sex	Age	Blood type	Rh	Race	Diagnosis	First consultation reason	Family background	Genetic Counseling	Disease Classification	FIX (%) technique: coagulometric reference values: 50-150%	PT (s) coagulometric method Reference values 9.9-11.8 seconds[2]	PTT (s) Coagulometric mehod. Reference values: 29-32 seconds[3]	Arthropathy
Am1B	Male	42	O	+	Caucasian	HB	Hemorrhages	Family history	no	severe	0,1	NA	NA	NA
Af1B	Female	49	B	+	Caucasian	carrier	NA	NA	NA	NA	NA	NA	NA	NA
Af1B *son	male	8	O	+	Caucasian	HB	Family history	cousins	no	moderate	1,5	11,1	38,1	NA
Af2B	Female	42	O	+	Caucasian	carrier	NA	NA	NA	NA	NA	NA	NA	NA
Af2B* son		10	O	+	Caucasian	HB	bruising	no	no	severe	0,4	NA	NA	NA

Patient code	Ristocetin cofactor (%)	Multimer analysis	RIPA (%)	Scheme of treatment	Administration	Factor type	Dose (UI)	Age of treatment start	Medicine brands	Inhibitor test result	Episodes of bleeding in the last year	Hemarthrosis	Occurrences of Hemarthrosis	Chronic Hemophilic Arthropathy
Am1B	NA	NA	NA	Prophylaxis	1 dose/week	Plasma derivative: FIX	1800	7	Immunine - Baxter	negative	10	yes	4	yes
Af1B	NA	NA	NA	NA	NA	NA	NA	NA	NA	NA	NA	NA	NA	no
Af1B *son	NA	NA	NA	on demand	NA	Recombinant	NA	2	Benefix factor recombinante IX	negative	3	no	NA	no
Af2B	NA	NA	NA	NA	NA	NA	NA	NA	NA	NA	NA	NA	NA	no
Af2B* son	NA	NA	NA	Prophylaxis	2 dose/week	Recombinant	1000	1	Benefix factor recombinante IX	negative	0	no	NA	no

**Patients not included in the study. Blood samples from their relatives with a carrier status were taken. NA: No answer. Factor level measurements shown in the table are only one measurement in time that corresponds to the last measurement taken in the medical history of the patients included in the study.*

[1]: **COAGULATION FACTOR IX (Christmas Factor, Antihemophilic Factor B):** Sample conditions included: Platelet-poor recentrifuged citrated plasma. 1 mL of plasma was separated and frozen immediately in a plastic tube. Sample, free of hemolysis and lipemia, was ship frozen on dry ice. The method used was the clot formation with a reference interval of 50 - 150%. Specifications of the assay: Automated single-layer clot performed on CS2100i coagulometer. Reagents: Calibrator plasma, normal control plasma, abnormal control plasma, FIX deficient plasma, PTT reagent: Actin FSL, Calcium chloride, owren buffer. Konkle, B., Huston, H., Nakaya, S. 2017. Synonyms: Christmas Disease, Factor IX Deficiency. Gene Reviews.

[2]: **PT (PROTHROMBIN TIME):** Sample conditions included: Platelet-poor recentrifuged citrated plasma. 1 mL of plasma was separated and frozen immediately in a plastic tube. Sample, free of hemolysis and lipemia, was ship frozen on dry ice. The method used was the clot formation. Specifications of the assay: Clot, automated performed on CS2100i coagulometer. Reagents: normal control plasma, abnormal control plasma, Innovin: recombinant thromboplastin (Siemens Healthcare. 2013. Guide insert).

[3]: **PTT (THROMBOPLASTIN PART TIME):** Sample conditions included: Platelet-poor recentrifuged citrated plasma. 1 mL of plasma was separated and frozen immediately in a plastic tube. Sample, free of hemolysis and lipemia, was ship frozen on dry ice. The method used was the clot formation with a reference interval of 9.9-11.82 seconds. Specifications of the assay: Clot, automated performed on CS2100i coagulometer. Reagents: Normal control plasma, abnormal control plasma, PTT reagent: Actin FSL, Calcium chloride. Siemens Healthcare. 2010. Guide insert.
