# Supplementary material for: Genetic and Bioinformatic Strategies to Improve Diagnosis in Three Inherited Bleeding Disorders in Bogotá, Colombia
Source: Genes (Basel). 2021 Nov 18;12(11):1807. doi: 10.3390/genes12111807 (PMC8625804; doi:10.3390/genes12111807)
Supplement: Supplementary file 1 [file genes-12-01807-s001.zip › Table S4.pdf]

Table S4. Phenotype characteristics of von Willebrand disease patients.

Patient code	Sex	Age	Blood type	Rh	Race	Diagnosis	First consultation reason	Family background	Genetic Counseling	Disease Classification	FVIII (%) technique: coagulometric reference values: 50-150%[1]	PT(s) coagulometric method Reference values 9.9-11.8 seconds[2]	PTT (s) Coagulometric mehod. Reference values: 29-32 seconds[3]	von Willebrand Factor (Antigen) technique: immunocapture reference values 50-160% [44]
Af1VW	Female	23	O	Positive	Caucasian	VWD type 2A, vitamin K deficiency and thrombocytopenia.	Hemorrhages, sudden bruising, difficulty in reconstructive surgeries. Non-compatible grafts.	Great aunt	no	VWD type 2A	168,5 (under prophylaxis treatment)	12,6	29,4	42,9
Af2VW	Female	38	O	Positive	Caucasian	VWD type 1	Heavy nosebleed Hemorrhages, hypermenorrhea Rectal bleeding	Mother presented hypermenorrhea	Yes: *F8* genetic analyses	VWD type 1	51,2	13	30,4	46,2
Af3VW	Female	19	A	Positive	Caucasian	VWD type 1	Hypermenorrhea, bruising, hemorrhages.	No family background	Sequencing of the *VWF gene*: no pathogenic variants are identified.	VWD type 1	87,6	NA	NA	59 (last data at the time of entering the study) 34.5 (data taken into account at the time of diagnosis)
Am1VW	Male	27	O	Positive	Caucasian	VWD type 2	Hemorrhages	No family background	no	VWD type 2N, but initially classified as HA.	2,7	11,6	54,7	56

Af5WD	Female	35	O	Positive	Caucasian	VWD type 1	Hypermenorrhea, hemorrhages.	No family background	no	VWD type 1	NA	NA	NA	40,2

NA: No answer. Factor level measurements shown in the table are only one measurement in time that corresponds to the last measurement taken in the medical history of the patients included in the study.

[1:] **COAGULATION FACTOR VIII (Antihemophilic Factor A):** Sample conditions included: Platelet-poor recentrifuged citrated plasma. 1 mL of plasma was separated and frozen immediately in a plastic tube. Sample, free of hemolysis and lipemia, was ship frozen on dry ice. The method used was the clot formation with a reference interval of 50 - 150%. Specifications of the assay: One-stage assay. Clot of an atapa, automated performed on a CS2100i coagulometer. Reagents: Calibrator plasma, normal control plasma, abnormal control plasma, FVIII deficient plasma, PTT reagent: Actin FSL, Calcium chloride, owren buffer.

[2:] **PT (PROTHROMBIN TIME):** Sample conditions included: Platelet-poor recentrifuged citrated plasma. 1 mL of plasma was separated and frozen immediately in a plastic tube. Sample, free of hemolysis and lipemia, was ship frozen on dry ice. The method used was the clot formation. Specifications of the assay: Clot, automated performed on CS2100i coagulometer. Reagents: normal control plasma, abnormal control plasma, Innovin: recombinant thromboplastin (Siemens Healthcare. 2013. Guide insert).

[3:] **PTT (THROMBOPLASTIN PART TIME):** Sample conditions included: Platelet-poor recentrifuged citrated plasma. 1 mL of plasma was separated and frozen immediately in a plastic tube. Sample, free of hemolysis and lipemia, was ship frozen on dry ice. The method used was the clot formation with a reference interval of 9.9-11.82 seconds. Specifications of the assay: Clot, automated performed on CS2100i coagulometer. Reagents: Normal control plasma, abnormal control plasma, PTT reagent: Actin FSL, Calcium chloride. Siemens Healthcare. 2010. Guide insert.

[4:] **VON WILLEBRAND ANTIGEN FACTOR (VWF:Ag):** Sample conditions included: Platelet-poor recentrifuged citrated plasma. 1 mL of plasma was separated and frozen immediately in a plastic tube. Sample, free of hemolysis and lipemia, was ship frozen on dry ice. The method used was Immunoturbidimetry, automated performed on a CS2100i coagulometer, with a reference interval of 50-160%. Reagents: Calibrator plasma, normal control plasma, abnormal control plasma, Von Willebrand Antigen measurement kit. Specifications of the assay: Haberichter, S. 2016. Von Willebrand factor propeptide: biology and clinical utility. Blood Journal. 126 (15). 1753-1761
