# Supplementary material for: Genetic and Bioinformatic Strategies to Improve Diagnosis in Three Inherited Bleeding Disorders in Bogotá, Colombia
Source: Genes (Basel). 2021 Nov 18;12(11):1807. doi: 10.3390/genes12111807 (PMC8625804; doi:10.3390/genes12111807)
Supplement: Supplementary file 1 [file genes-12-01807-s001.zip › Table S5.pdf]

Table S5. Phenotype characteristics of von Willebrand disease patients.

Patient code	Ristocetin cofactor activity (%) technique: Aggregometry, Immunoturbidimetry with a reference interval of Blood Group O: 53.0 - 152.8 and Blood Groups A, B, AB: 71.2 - 178.5[1]	Scheme of treatment	Administration	Factor type	Dose (UI)	Age of treatment initiation	Medicine brands	Inhibitor test result	Episodes of bleeding in the last year	Hemarthrosis	Occurrences of Hemarthrosis	Chronic Hemophilic Arthropathy
Af1VW	72,7	Prophylaxis/ on demand	NA	Recombinant - Vitamin K1 - Desmopressin	NA	10	Baxter (factor), Konakion (vitamin K)	-	3	Yes	3	No
Af2VW	44	on demand	Monthly/Each period	Tranexamic acid, currently Mirena	NA	22	NA	-	21	No	0	No
Af3VW	43,7	on demand	NA	NA	NA	NA	NA	-	48	No	0	No
Am1VW	55,1	Prophylaxis	3 time/week	Recombinant - Plasma derivative	(1000 UI FVIII + 2400 UI FVW)	22	HAEMATE P	-	6	yes	6	yes
Af4VW	NA	No treatment	NA	NA	NA	NA	NA	-	7	No	0	No

NA: No answer.

[1]: **FUNCTIONAL VON WILLEBRAND FACTOR: Activity (VWF: Ac), Ristocetin Cofactor (VWF: Rco):** Sample conditions included: Platelet-poor recentrifuged citrated plasma. 1 mL of plasma was separated and frozen immediately in a plastic tube. Sample, free of hemolysis and lipemia, was ship frozen on dry ice. The method used was Aggram of Helena Laboratories with a reference interval of Blood Group O: 53.0 - 152.8 and Blood Groups A, B, AB: 71.2 - 178.5. Reagents: Calibrator plasma, normal control plasma, abnormal control plasma, Ristocetin, normal platelets fixed in formalin, owren buffer. Specifications of the assay: Federici, A.B. 2016. Current and emerging approaches for assessing von Willebrand disease in 2016. International Journal of Laboratory Hematology.38 (1). 41-49
