# Supplementary figures and images for: Genetic and Bioinformatic Strategies to Improve Diagnosis in Three Inherited Bleeding Disorders in Bogotá, Colombia
Source: Genes (Basel). 2021 Nov 18;12(11):1807. doi: 10.3390/genes12111807 (PMC8625804; doi:10.3390/genes12111807)

Sample - melt curve

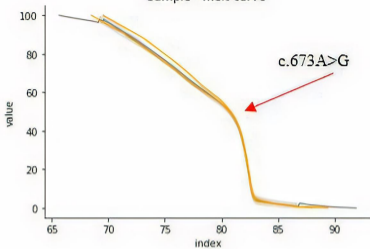

Sample- negative derivative curve

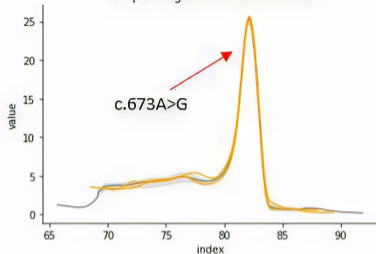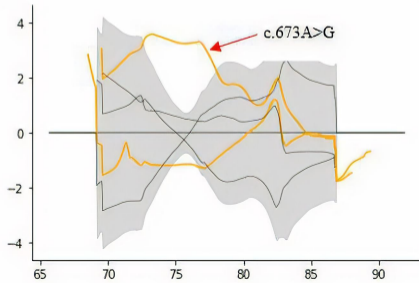

Supplement: Supplementary file 1 [file genes-12-01807-s001.zip › Figure S1.pdf]

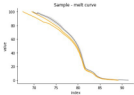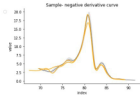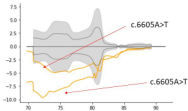

Supplement: Supplementary file 1 [file genes-12-01807-s001.zip › Figure S2.pdf]

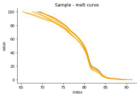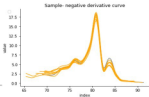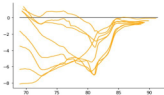

Supplement: Supplementary file 1 [file genes-12-01807-s001.zip › Figure S3.pdf]
